# Significant functional improvements and radiographic stability following fully cemented total hip arthroplasty in elderly patients: A 2-year prospective study

**DOI:** 10.1016/j.jor.2025.07.027

**Published:** 2025-08-05

**Authors:** Carlo Marega, Domenico De Mauro, Daniele Lamberti, Giuseppe Rovere, Francesco Bosco, Luca Marega

**Affiliations:** a Department of Orthopedic Surgery, Ospedale San Camillo, 38122, Trento, Italy; b Department of Orthopedics and Geriatrics Sciences, Università Cattolica del Sacro Cuore, 00168, Rome, Italy; c Orthopedic Unit, Department of Public Health, Federico II University, 80131, Naples, Italy; d Department of Clinical Science and Translational Medicine, Section of Orthopaedics and Traumatology, University of Rome “Tor Vergata”, 00133, Rome, Italy; e Department of Precision Medicine in Medical, Surgical and Critical Care (Me.Pre.C.C.), University of Palermo, 90133, Palermo, Italy; f Department of Orthopaedics and Traumatology, G.F. Ingrassia Hospital Unit, ASP 6, 90131, Palermo, Italy

**Keywords:** Total hip arthroplasty, Cemented stem, Cemented cup, Outcomes, Elderly

## Abstract

**Purpose:**

Using fully cemented total hip arthroplasty (THA), involving both a cemented cup and stem, remains uncommon in several countries. This study aimed to evaluate the clinical and radiographic outcomes of fully cemented primary THA in an elderly population.

**Methods:**

This prospective, single-center, longitudinal cohort study enrolled patients in THA. Clinical assessments included the Harris Hip Score (HHS), the Timed Up-and-Go (TUG) test, and the Hip Disability and Osteoarthritis Outcome Score (HOOS). Radiographic evaluation was performed using anteroposterior and lateral X-rays to assess implant positioning and stability.

**Results:**

Forty patients completed the 2-year follow-up (FU), with a mean age of 79.0 ± 6.0 years. Clinical outcomes demonstrated significant improvements at 2-year FU: the mean HHS increased from 42 ± 15 preoperatively to 93 ± 9; TUG improved from 24 ± 25 s preoperatively to 8 ± 3 s; and HOOS improved from 35 ± 14 preoperatively to 84 ± 17. No intraoperative complications or implant-related adverse events were reported during the 2-year follow-up.

**Conclusion:**

This study highlights substantial improvements in functional outcomes, patient satisfaction, and radiographic evidence of good implant stability at 2-year follow-up. These findings support fully cemented THA as a safe and effective treatment option for elderly patients.

**Level of evidence:**

IV.

## 1 Introduction

Total hip arthroplasty (THA) has been celebrated as the “surgery of the century” due to its transformative impact on the treatment of hip osteoarthritis (OA).^1^ The first THA, pioneered by Sir John Charnley in the 1960s, introduced cemented implants, which became a cornerstone in orthopedic surgery.^2^ Despite the success of this approach, the development of uncemented implants in the 1970s represented a significant evolution, driven by the promise of easier reproducibility and concerns over the so-called “cement disease”.^3^ This condition, characterized by aseptic loosening and osteolysis at the bone-implant interface, was initially attributed to cement use. However, subsequent research identified polyethylene and metal wear particles as the primary contributors to aseptic loosening rather than the cement itself.^4^^,^^5^

The trend toward cementless THA grew during the 1980s, with adoption rates varying significantly across countries.^6^^,^^7^ Fully cemented THA remains relatively uncommon in nations such as Italy (3.4 % over the last 15 years),^8^ the United States (4.55 % in 2022),^9^ and Australia (1.9 %, with 36.2 % of hybrid implants in 2022).^10^ In contrast, its usage is more prevalent in countries like the United Kingdom (19.1 % of all THAs in 2022)^11^ and Sweden (52 % in 2022),^12^ although a slight decline has been observed in recent years.

The development of cementless implants aimed to reduce aseptic loosening and improve surgical reproducibility, but these goals have only been partially achieved. Accumulating evidence has shown that aseptic loosening is more closely related to polyethylene wear than to cement use.^13^, ^14^, ^15^, ^16^, ^17^

National joint registries from countries such as the UK, Australia, and Sweden consistently report lower revision rates and excellent long-term outcomes for cemented implants, particularly in elderly populations.^10^, ^11^, ^12^, ^14^, ^16^

This study aimed to evaluate the clinical and radiographic outcomes of fully cemented primary THA, specifically using Mueller cemented cups and short cemented stems. Additionally, the study aimed to assess early functional improvements, patient satisfaction, implant survivorship, and the incidence of complications within a short-term follow-up period.

## 2 Material and methods

### 2.1 Study design and ethical approval

This prospective observational study was conducted at the Department of Orthopedic Surgery, Ospedale San Camillo, Trento, TN, Italy, between January 2022 and December 2022. Patients undergoing primary THA were identified from the hospital registry and assessed for eligibility. Informed consent was obtained from all participants before inclusion in the study. The study adhered to the ethical principles of the Declaration of Helsinki and was approved by the Local Ethics Committee (Protocol No. H-31/20).

### 2.2 Patient selection

The study population was selected to ensure homogeneity and clinical relevance. Inclusion and exclusion criteria determined eligibility. Patients were included if they were aged 65 years or older, presented with hip conditions necessitating surgical intervention—such as primary or secondary osteoarthritis, avascular necrosis, rheumatoid arthritis, or femoral neck fractures—and required THA to alleviate pain or improve joint function. Exclusion criteria were active or suspected infections, hypersensitivity or allergies to the implant materials, significant neurological or musculoskeletal disorders that could interfere with functional recovery, or incomplete clinical data resulting from dropout during follow-up. Each patient was systematically screened, and those meeting all criteria were enrolled consecutively into the study.

### 2.3 Implant features

The implants used were the Friendly Short femoral stem, and the Mueller cemented cup from Lima Corporate (Italy).

The Friendly Short stem is an 80 mm monolithic, polished, collarless implant with a rectangular cross-section and rounded rims, designed to optimize load transfer and minimize stress shielding, particularly in elderly patients. Its straight geometry enhances cement mantle integrity, crucial for long-term stability.^18^

The Mueller cemented cup, made of ultra-high-molecular-weight polyethylene (UHMWPE), features an equatorial radiopaque metal ring for accurate radiographic assessment of cup orientation and positioning.

Cups were available in standard and protruded designs for anatomical customization. A modular cobalt-chromium-molybdenum (CoCrMo) femoral head was used to ensure compatibility and durability.

### 2.4 Surgical technique

All procedures were performed by the senior surgeon (L.M.), ensuring consistency and reproducibility. Spinal subarachnoid anesthesia was used to optimize hemodynamic control and recovery.

A modified Gibson-Moore posterolateral approach was employed with the patient in lateral decubitus, allowing superior access to the acetabulum and femur.^18^^,^^19^ The approach involved careful dissection of the gluteus maximus, short external rotators, and capsule, followed by precise implant placement.

A fourth-generation cementing technique was meticulously applied, involving thorough bone preparation, retrograde insertion of low-viscosity PMMA cement, and pressurization to ensure uniform distribution and strong bone-cement interlock.^18^ Intraoperative verification of acetabular orientation and stem alignment was performed to optimize biomechanical restoration.

### 2.5 Postoperative assessment

Clinical and radiographic evaluations were performed preoperatively and at 6 weeks, 1 year, and 2 years post-surgery.

Clinical outcomes were assessed using the Harris Hip Score (HHS),^20^ the Timed Up-and-Go (TUG) test,^21^ and the Hip Disability and Osteoarthritis Outcome Score (HOOS),^21^^,^^22^ providing a comprehensive overview of functional recovery and quality of life.

Radiographic assessments included AP pelvis and lateral hip X-rays to evaluate implant positioning, stability, and potential complications. Radiolucent lines were classified according to DeLee-Charnley^23^ for the acetabulum and Gruen^24^ for the femoral stem.

Heterotopic ossifications were graded using the Brooker classification.^25^ Clinical symptoms were correlated with radiographic findings to detect early complications.

Adverse events, including surgical, implant-related, or systemic complications, were systematically recorded, ensuring a robust analysis of functional outcomes and implant stability.

### 2.6 Statistical analysis

Data were collected using an ad hoc electronic case report form (e-CRF) designed by the clinical study protocol. Statistical analysis was performed using R software (version 4.3, R Foundation for Statistical Computing, Vienna, Austria), with Microsoft® Excel 365 used for initial data management and organization. Descriptive statistics were employed to summarize preoperative, intraoperative, and postoperative variables. Continuous variables were expressed as mean, standard deviation (SD), and median values, while categorical variables were presented as absolute and relative frequencies. The Student's t-test was applied to evaluate differences in continuous variables, such as functional scores and mobility metrics. Categorical data, including complications and events, were analyzed using the appropriate Chi-squared or Fisher's exact test. Survival analysis was conducted using Kaplan-Meier curves to estimate implant survival rates over the follow-up period. Differences in survival distributions between groups were assessed using the log-rank test. Implant failure was defined as the need for revision surgery due to any cause. A significance level of p < 0.05 was used to determine statistical significance.

## 3 Results

### 3.1 Patients’ selection and characteristics

**Fig. 1:**
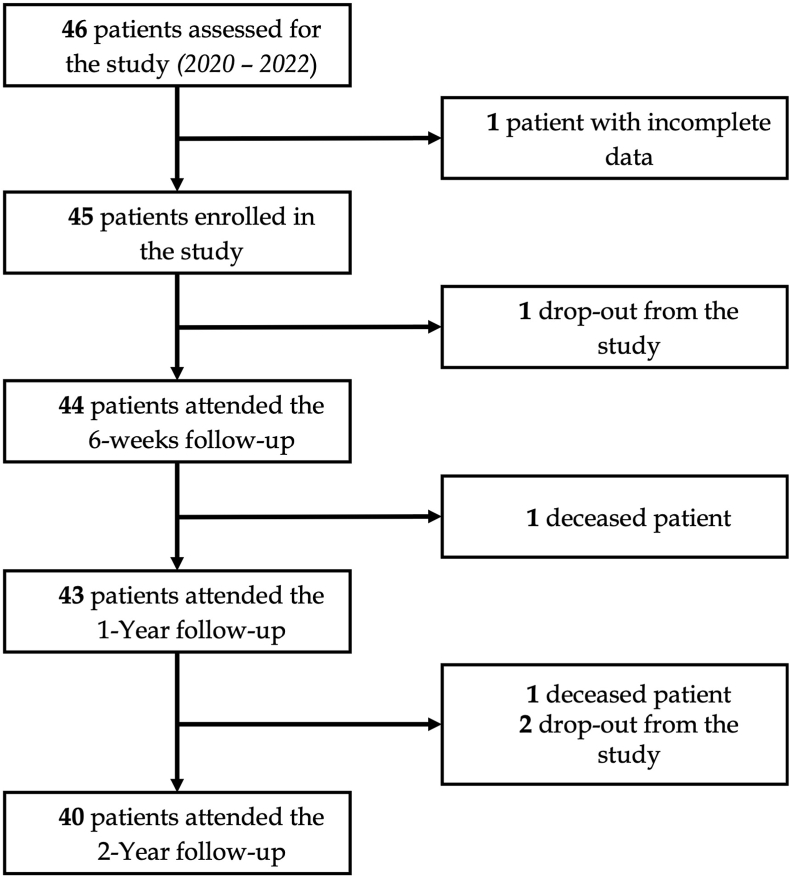
Patients' selection flowchart that illustrates the process of patient screening, inclusion, and exclusion, along with the number of patients assessed, enrolled, and completing the study up to the 2-year follow-up.

A total of 46 patients were screened for eligibility following the provision of written informed consent, of whom 45 were ultimately enrolled in the study. Patient enrollment began on March 1, 2022, and concluded on October 25, 2022. The selection process, including inclusion and exclusion criteria, is outlined in the flowchart provided in Fig. 1. Demographic characteristics and implant specifications are summarized in Table 1.

**Table 1:** Demographics and baseline characteristics of study population.

Demographics	Mean ± SD
Weight (kg)	70 ± 12
Height (cm)	165 ± 8
BMI (kg/m^2^)	26 ± 4
Age (years)	79 ± 6
Diagnosis	n. (%)
Primary OA	40 (88.9 %)
Rheumatoid arthritis	1 (2.2 %)
Post-traumatic OA	1 (2.2 %)
Fracture of the femoral neck	1 (2.2 %)
ANFH	1 (2.2 %)
Affected side	n. (%)
Right	20 (44.4 %)
Left	25 (55.6 %)
Trendelenburg sign	n. (%)
Positive	36 (80.0 %)
Negative	9 (20.0 %)
Implants	n. (%)
Cup	
Standard	32 (71.0 %)
Protruded	13 (29.0 %)
Stem	
Standard	40 (88.9 %)
Lateralized	5 (11.1 %)

SD: Standard Deviation; BMI: Body Mass Index; OA: Osteoarthritis; ANFH: Avascular Necrosis of the Femoral Head.

n: Number of patients; %: percentage; kg: Kilograms; cm: Centimeters; m: Meters.

All patients received the Friendly Short femoral stem, equipped with a distal centralizer to ensure proper alignment and stability. Acetabular cup and femoral stem sizes were selected based on individual anatomy, with a tailored approach supported by preoperative planning. Metal femoral heads with a 12/14 taper and a 32 mm diameter were used consistently across the cohort, with head sizes adapted as appropriate.

### 3.2 Postoperative assessment: clinical evaluation

**Fig. 2:**
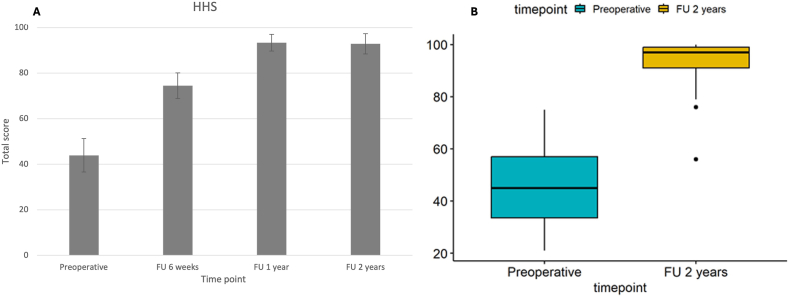
Harris Hip Score (HHS) results. (A) Bar graph representation of the total HHS measured at preoperative baseline, 6 weeks, 1 year, and 2 years post-surgery, highlighting significant functional improvement over time. (B) Box plot representation comparing total HHS results at preoperative baseline and 2-year follow-up, showing the distribution and variability of scores. HHS: Harris Hip Score; FU: Follow-up.

Clinical outcomes demonstrated significant and sustained improvement following surgery. The HHS showed a marked increase from the preoperative assessment to the 2-year follow-up, reflecting stable functional recovery over time (Table 2, Fig. 2). Notably, one patient with an initially excellent 1-year result experienced a decline at 2 years due to mobilization of a contralateral acetabular component (implanted prior to this study), requiring revision surgery shortly afterward.

**Table 2:** Change in total Harris Hip Score (HHS), Time Up-and-Go test (TUG), and Hip Disability and Osteoarthritis Outcome Score (HOOS) score from preoperative (baseline) to 6 weeks*,* 1 year and 2 years follow-up.

Time point	Mean ± SD	Min	Max	Median	p-value (preoperative vs postoperative)
HHS results					
Preoperative (n = 45)	43.9 ± 14.7	16	75	44	–
6 weeks FU (n = 44)	74.5 ± 11.3	50	99	75.5	<0.001
1 year FU (n = 43)	93.4 ± 7.3	72	100	97	<0.001
2 years FU (n = 40)	92.9 ± 8.9	56	100	97	<0.001
TUG results					
Preoperative (n = 45)	24.1 ± 24.8	6	152	17	–
6 weeks FU (n = 44)	8.8 ± 2.6	5	18	8	<0.001
1 year FU (n = 43)	8.0 ± 3.1	5	18	7	<0.001
2 years FU (n = 40)	7.9 ± 3.4	4	17	7	<0.001
HOOS score					
Preoperative (n = 45)	34.9 ± 14.1	9	77	33	–
6 weeks FU (n = 44)	76.0 ± 13.2	45	93	78	<0.001
1 year FU (n = 43)	84.8 ± 13.9	51	100	90	<0.001
2 years FU (n = 40)	84.1 ± 17.2	33	100	89.5	<0.001

HHS: Harris Hip Score; FU: Follow-Up; TUG: Timed Up and Go; HOOS: Hip disability and Osteoarthritis Outcome Score; SD: Standard Deviation; Min: Minimum; Max: Maximum; Median: Median; n: Number of patients; vs: versus.

Timed Up and Go (TUG) test performance improved rapidly by 6 weeks postoperatively and remained stable through subsequent follow-ups, indicating early restoration of mobility. Similarly, the Hip Disability and Osteoarthritis Outcome Score (HOOS) showed substantial gains, underscoring meaningful improvements in pain relief, functional capacity, and quality of life (Table 2).

### 3.3 Postoperative assessment: radiological evaluation

Radiographs showed a mean leg-length discrepancy of 7.0 ± 12.0 mm. Most patients (68.9 %, n = 31) had no varus/valgus deformity; 13.3 % (n = 6) had valgus and 17.8 % (n = 8) varus deformity.

The mean acetabular abduction angle was 44.1° ± 4.2 (range 30°–55°), remaining stable over time. Heterotopic ossifications were absent in 80 % (n = 36), with Class I in 15.6 % (n = 7) and Class II in 4.4 % (n = 2), without progression at 2 years.

No radiolucent lines were observed immediately postoperatively or at 6 weeks. At 1 year, 25.6 % (n = 11) showed 1 mm radiolucency in Zone 1 (DeLee-Charnley), 2.3 % (n = 1) in Zone 2; at 2 years, 30 % (n = 12) in Zone 1 and 5 % (n = 2) in Zone 2, with two patients exhibiting lines in both Zones 1 and 2. These minor changes indicated stable implants.

The mean postoperative angle between diaphysis and stem was 1.5° ± 2.7 (range 0°–10°), consistent across follow-ups, reflecting stable stem positioning.

No radiolucent lines or osteolysis were found in any Gruen zone. Comprehensive assessments revealed no loosening, subsidence, or implant instability over 2 years, confirming durable fixation and cemented component reliability.

### 3.4 Adverse events reporting

A total of 17 adverse events were recorded during the study, as detailed in Table 3. Among these, seven were classified as serious adverse events. Significantly, no serious events were directly attributed to the implanted medical device. However, four events were associated with the surgical procedure under investigation, with one deemed probably related and three considered possibly related. These findings underscore the critical need for meticulous intraoperative and postoperative management to mitigate risks while affirming the safety profile of the implanted components.

**Table 3:** Adverse events/serious adverse events categorization.

AE/SAE general category	n	(%)	Specific category	n	(%)
Injury and procedural complications	4	(23)	Trauma with periprosthetic femoral fracture	1	(25)
			Postoperative anemia	3	(75)
Cardiac disease	3	(18)	Heart failure	1	(33)
			Postoperative alteration of the rhythm	1	(33)
			Atrial fibrillation	1	(33)

Musculoskeletal disorder	7	(41)	Osteoarthritis	1	(14)
			Trochanteritis	2	(29)
			Arthrosis	1	(14)
			Mobilization of a contralateral implant	1	(14)
			Degenerative flat foot	1	(14)
			Postoperative dislocation	1	(14)

Tumor	1	(6)	–	–	–

Nervous system disorder	1	(6)	–	–	–

Not known	1	(6)	–	–	–

AE: Adverse Event; SAE: Serious Adverse Event; n: Number of patients; %: percentage.

### 3.5 Device survivorship analysis

The endpoint for survivorship analysis was the revision of the acetabular component for any reason. No revisions occurred during the 2-year follow-up, resulting in a 100 % survivorship rate for the Mueller cemented cup.

One revision among 40 cases (97.9 % survivorship; 95 % CI: 93.3 %–100 %) was recorded for the femoral component due to a periprosthetic fracture after a high-energy skiing accident 9 months postoperatively unrelated to implant design or material.

**Fig. 3:**
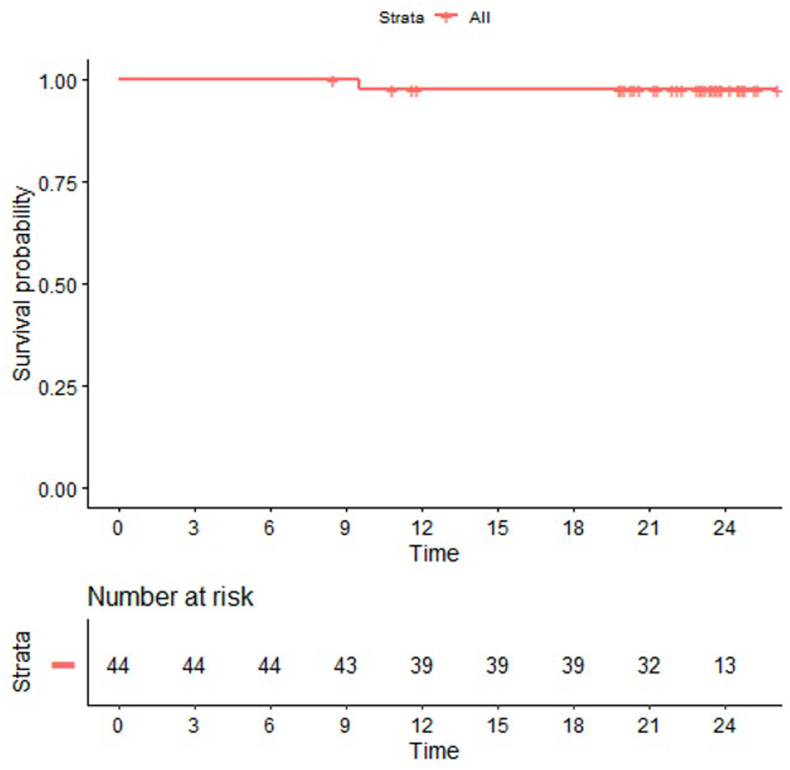
The Kaplan-Meier survival curve of femoral side components illustrates the cumulative survival probability of the femoral components over the 2-year follow-up period, demonstrating a survivorship rate of 97.9 % at the study endpoint.

Fig. 3 shows the Kaplan-Meier curve for the femoral stem, confirming the high durability of the implant system over two years.

## 4 Discussion

### 4.1 Main findings

The main finding of this study is that Mueller cemented cups combined with cemented Friendly Short stems provide excellent clinical, radiographic, and safety outcomes in elderly patients undergoing THA. Over a 2-year follow-up, a substantial improvement in HHS of 49 points was observed, exceeding the threshold for moderate improvement^26^ and matching or surpassing results typically achieved with cementless systems, even in younger populations.^27^ Additionally, radiographic analysis confirmed implant stability, with no evidence of osteolysis, loosening, or significant radiolucent lines, and no adverse events were directly related to the implants. These findings underscore the reliability and effectiveness of fully cemented THA in a geriatric population.

### 4.2 Consistency with prior findings

The functional outcomes observed in this study are consistent with, and in some aspects superior to, those previously reported for cemented THA in elderly patients. Prior studies, including those by Haverkamp et al., Johanson et al., and Kristof et al., have highlighted favorable long-term functional recovery after cemented hip arthroplasty.^28^, ^29^, ^30^ Despite differences in patient age and follow-up duration, our cohort showed early recovery patterns comparable to or better than those reported in younger or mixed-age populations, reinforcing the effectiveness of cemented implants in older patients. Similarly, mid-term studies such as those by Duarte et al. and Jonsson et al. support the robust clinical outcomes achievable with this construct.^31^^,^^32^

### 4.3 Radiographic outcomes

Radiographic findings further confirmed implant stability, with no evidence of stem loosening, osteolysis, or clinically significant radiolucencies. The minor acetabular radiolucent lines occasionally observed remained stable over time and were not associated with symptoms or complications, underscoring the mechanical reliability of the system in the short term.

### 4.4 Clinical implications

In terms of patient-reported outcomes (PROMs), we observed marked improvements in pain relief, functional capacity, and quality of life, as reflected in HOOS scores. These results are in line with prior reports and highlight the potential of cemented THA to deliver rapid recovery and durable early outcomes in elderly patients. Importantly, our study adds prospective evidence focusing on this specific population, complementing the insights provided by large-scale registry data.

Despite the declining use of fully cemented THA in favor of cementless systems, this study reaffirms the value of cemented implants for older patients. Cemented systems provide immediate mechanical stability, crucial for individuals with osteoporosis or poor bone quality, unlike cementless systems that require biological fixation over time.

There remains a paucity of prospective studies specifically examining fully cemented THA in elderly patients using modern cementing techniques and contemporary implant designs. Most existing studies either focus on older implant generations or aggregate cemented and cementless outcomes without clear differentiation.^28^, ^29^, ^30^, ^31^, ^32^

National joint registries^10^, ^11^, ^12^ and multicenter reviews^16^ often lack detailed stratification by cementation status or stem design, limiting insight into the benefits of fully cemented constructs. Moreover, longitudinal PROMs assessment in cemented THA remains underreported,^22^^,^^31^, ^32^, ^33^ and early minor radiographic changes are seldom correlated with clinical outcomes,^23^^,^^24^ leaving gaps in predicting implant longevity.

This prospective study offers novel evidence on fully cemented THA in elderly patients (mean age 79), using modern cementing techniques, a polished short stem, and a cemented Mueller cup. A 49-point HHS gain and significant TUG and HOOS improvements matched or exceeded outcomes in younger, cementless-treated populations.^27^, ^28^, ^29^ Radiographically, no pathological loosening and only minimal, non-clinically relevant radiolucencies were observed,^23^^,^^24^ confirming early mechanical stability. These findings underscore the effectiveness and safety of cemented THA in the elderly, a group often underrepresented despite reduced bone quality making them ideal candidates.

### 4.5 Study strengths

The study's strengths include its strict elderly cohort, prospective design, and comprehensive clinical, radiographic, and PROMs-based evaluation, providing robust and timely data to guide implant selection and clinical decision-making.

Although our study does not introduce novel surgical techniques, implant designs, or evaluation methods, it contributes complementary prospective evidence supporting the effectiveness and safety of fully cemented THA in elderly patients. These findings align with previously reported outcomes from national registries and long-term studies, reinforcing the continued relevance of cemented constructs in this population, particularly in healthcare settings where their use remains limited. While registry data provide valuable large-scale, long-term insights, our study contributes complementary prospective evidence focusing on early clinical, functional, and radiographic outcomes, particularly in an elderly Italian cohort—a group underrepresented in prior prospective analyses.

### 4.6 Study limitations

Several limitations must be acknowledged. First, the single-center design and the fact that all procedures were performed by one senior surgeon limit the generalizability of the findings, as outcomes may reflect local surgical expertise, patient selection, perioperative protocols, or institutional practices rather than implant characteristics alone. Conducting multicenter studies would enhance external validity and allow broader comparisons across healthcare systems. Furthermore, the inclusion criteria and the requirement for adherence to all follow-up visits likely favored the selection of healthier elderly patients with better baseline functional status, potentially overestimating the generalizability of the functional outcomes to a broader, more comorbid elderly population. Second, although the study was adequately powered to detect significant changes in primary clinical outcomes, the relatively small sample size (n = 40) and absence of a formal power calculation reduce the statistical strength and external validity of the findings. Moreover, the limited cohort size precluded meaningful subgroup analyses (e.g., by age strata, comorbidities, or implant characteristics). A larger, multi-institutional cohort would allow for more robust analyses and generalizable conclusions. Additionally, the statistical analysis was primarily descriptive, without multivariate adjustment for potential confounders such as BMI, comorbidities, or ASA grade, limiting the ability to identify independent predictors of clinical and radiographic outcomes. Furthermore, although improvements in HHS, HOOS, and TUG were statistically significant, they were not formally contextualized against minimal clinically important difference (MCID) thresholds, nor adjusted for baseline functional heterogeneity. This limitation was primarily due to the exploratory nature of the study and the relatively small sample size, which was not designed or powered to perform stratified analyses or to robustly estimate clinically meaningful changes across different patient profiles. Third, although the 2-year follow-up period provides valuable short-term data, it does not allow for conclusions on long-term implant survivorship, wear, or late complications. While national registries and multicenter studies have already provided robust long-term evidence on cemented implants, our study was specifically designed to capture early functional improvements, radiographic stability, and safety profiles, which are often underreported but crucial for optimizing immediate postoperative management and patient counseling. Furthermore, the assessment of radiographic outcomes was limited to descriptive evaluation, without quantitative grading or blinded independent review, and no advanced imaging modalities (e.g., radiostereometric analysis or computed tomography) were used to detect micromotion or early signs of loosening. The observed radiolucent lines in Zone 1 at 2 years, although considered non-clinically relevant in this context, cannot be confidently interpreted as benign in the absence of longer-term follow-up and more precise radiographic assessment. Additionally, although 17 adverse events were reported, the lack of detailed stratification by severity, timing, and follow-up management limits the interpretability of complication data. Furthermore, the impact of contralateral hip revision in one patient on functional and quality-of-life outcomes was not adequately analyzed, potentially influencing the aggregated patient-reported outcome scores. Finally, the absence of randomization, blinding, and a direct comparison group critically limits the ability to draw causal or comparative conclusions regarding the relative performance of fully cemented THA versus cementless or hybrid constructs. Although we referenced data from national registries and prior literature, our observational design provides preliminary evidence rather than definitive comparative or causal claims. Therefore, further randomized controlled trials or matched cohort studies are required to validate and extend these findings. Moreover, the study was fully funded by LimaCorporate S. p.A., and one senior author serves as a consultant for the company. While the use of a single implant brand reflects our institutional standard of care, it may raise concerns about generalizability and potential selective reporting. We acknowledge that no independent oversight or third-party data verification was employed, which may introduce bias despite the use of validated clinical and radiographic outcome measures. Future studies should incorporate independent monitoring and consider multi-brand designs to enhance generalizability and minimize sponsorship-related bias.

### 4.7 Future research directions

Future research should aim to address these limitations by incorporating matched cohort designs or randomized comparisons with cementless or hybrid constructs, allowing for more robust comparative analyses. Extending follow-up to at least 5–10 years will be crucial to assess mid-to long-term outcomes, particularly related to aseptic loosening, polyethylene wear, and implant survivorship. Moreover, multicenter collaborations involving multiple surgeons and diverse patient populations would enhance generalizability and reduce potential biases associated with single-center or single-surgeon series. Applying multivariate statistical analyses could help identify independent predictors of clinical, functional, and radiographic outcomes, offering deeper insights into patient- and implant-related factors. Finally, strengthening radiographic evaluations with validated objective tools such as radiostereometric analysis or advanced imaging modalities may improve the precision of implant stability assessments and inform early detection of potential failure mechanisms.

## 5 Conclusion

The results of this study demonstrate that Mueller cemented cups and cemented Friendly Short stems offer excellent clinical, radiographic, and patient-reported outcomes in elderly patients undergoing total hip arthroplasty. The substantial improvement in functional outcomes, radiographic stability, and absence of device-related adverse events underscore the reliability and effectiveness of fully cemented systems in this population. These findings suggest that fully cemented THA remains a valuable and underutilized option, particularly for elderly patients, and should be considered a viable alternative to cementless systems in appropriately selected cases.

## Guardian/patient's consent

Not Applicable.

## Credit author statement

CM and LM contributed to the study conception and design. Material preparation and data collection were performed by DL and CM. Data analyses were performed by DDM and GR. The first draft of the manuscript was written by CM, and DDM and all authors commented on previous versions of the manuscript. LM and FB assessed the scientific contents and the writing. All authors read and approved the final manuscript.

## Ethical statement

The research protocol was approved by the local Institutional Review Board (H-31/20).

## Funding STATEMENT

This work has been funded by the company LimaCorporate S. p.A, Villanova di S. Daniele del Friuli (UD), Italy.

## Conflict of interest statement

Luca Marega, MD, receives speaker and consultant honoraria from LimaCorporate S. p.A. All the other authors have no relevant financial or non-financial interests to disclose.
